# Histologic chorioamnionitis in preterm infants: correlation with brain magnetic resonance imaging at term equivalent age

**DOI:** 10.1186/s12887-018-1001-6

**Published:** 2018-02-15

**Authors:** Claire Granger, Alicia J. Spittle, Jennifer Walsh, Jan Pyman, Peter J. Anderson, Deanne K. Thompson, Katherine J. Lee, Lee Coleman, Charuta Dagia, Lex W. Doyle, Jeanie Cheong

**Affiliations:** 1 Department of Neonatal Medicine, Royal Children’s Hospital, Melbourne, Australia; 2 Victorian Infant Brain Studies, Murdoch Childrens Research Institute, Melbourne, Australia; 3 Department of Physiotherapy, University of Melbourne, Melbourne, Australia; 4 Neonatal Services, Royal Women’s Hospital, Melbourne, Australia; 5 Department of Obstetrics & Gynaecology, University of Melbourne, Melbourne, Australia; 6 Paediatric, Infant, Perinatal Emergency Retrieval, Royal Children’s Hospital, Melbourne, Australia; 7 Department of Pathology, Royal Women’s Hospital, Melbourne, Australia; 8 Monash Institute of Cognitive & Clinical Neurosciences, Monash University, Melbourne, Australia; 9 Department of Paediatrics, University of Melbourne, Melbourne, Australia; 10 Developmental Imaging, Murdoch Childrens Research Institute, Melbourne, Australia; 11 Florey Institute of Neuroscience and Mental Health, Melbourne, Australia; 12 Clinical Epidemiology and Biostatistics, Murdoch Childrens Research Institute, Melbourne, Australia; 13 Department of Radiology, Royal Children’s Hospital, Melbourne, Australia; 14 Neonatal Intensive Care Unit, Princess Royal Maternity Hospital, 16 Alexandra Parade, Glasgow, G31 2ER Scotland

**Keywords:** Chorioamnionitis, Preterm, Magnetic resonance imaging, Intraventricular haemorrhage, Brain volumes

## Abstract

**Background:**

To explore the associations between histologic chorioamnionitis with brain injury, maturation and size on magnetic resonance imaging (MRI) of preterm infants at term equivalent age.

**Methods:**

Preterm infants (23–36 weeks’ gestational age) were recruited into two longitudinal cohort studies. Presence or absence of chorioamnionitis was obtained from placental histology and clinical data were recorded. MRI at term-equivalent age was assessed for brain injury (intraventricular haemorrhage, cysts, signal abnormalities), maturation (degree of myelination, gyral maturation) and size of cerebral structures (metrics and brain segmentation). Histologic chorioamnionitis was assessed as a predictor of MRI variables using linear and logistic regression, with adjustment for confounding perinatal variables.

**Results:**

Two hundred and twelve infants were included in this study, 47 (22%) of whom had histologic chorioamnionitis. Histologic chorioamnionitis was associated with higher odds of intraventricular haemorrhage (odds ratio [OR] (95% confidence interval [CI]) = 7.4 (2.4, 23.1)), less mature gyral maturation (OR (95% CI) = 2.0 (1.0, 3.8)) and larger brain volume (mean difference in cubic centimeter (95% CI) of 14.1 (1.9, 26.2)); but all relationships disappeared following adjustment for perinatal variables.

**Conclusion:**

Histologic chorioamnionitis was not independently associated with IVH, less mature gyral maturation or brain volume at term-equivalent age in preterm infants.

**Electronic supplementary material:**

The online version of this article (10.1186/s12887-018-1001-6) contains supplementary material, which is available to authorized users.

## Background

The developing brain is vulnerable and is subject to a number of antenatal and perinatal stressors. Rapid brain growth and development occurs during the later stages of pregnancy, and any insult to the developing brain during this time may result in adverse neurodevelopmental sequelae [1].

Chorioamnionitis has been implicated as an antecedent of spontaneous preterm labour [2]. Chorioamnionitis refers to inflammation of the placental tissues [3], and is thought to result from ascending infection into the uterus from the vagina. Pathogens are usually of low virulence and are difficult to culture using traditional methods [3]. Chorioamnionitis is defined as histologic, clinical, or both. The former is considered the “gold standard” [4], whereas the definition for clinical chorioamnionitis is inconsistent and has been shown to correlate poorly with histologic changes in the placenta [4]. Therefore the focus of this study was on histologic chorioamnionitis.

A number of perinatal variables are associated with chorioamnionitis, including earlier preterm delivery, prolonged rupture of membranes, low birthweight and maternal peripartum fever [1, 2, 5]. However, many studies of chorioamnionitis do not include infants born moderate to late preterm (MLPT; 32–36 weeks’ gestation). Understanding the effects of chorioamnionitis across the gestational age spectrum is important given the majority of preterm births are moderate or late preterm [6].

Inflammation, including chorioamnionitis, has been implicated in the pathogenesis of brain injury in preterm children. The potential mechanisms include pro-inflammatory cytokine release related to the fetal inflammatory response [7], which, in experimental models, has resulted in brain alterations consistent with impaired myelination, neuronal loss and white matter lesions [8].

Previous studies have attempted to establish a link between chorioamnionitis, and subsequent neurodevelopment and brain abnormalities. The associations between chorioamnionitis, and cystic periventricular leukomalacia and cerebral palsy are well documented. [9] However, the associations between chorioamnionitis and intraventricular haemorrhage, brain volumes and brain maturation are less consistent [10–12].

To address research gaps surrounding chorioamnionitis, we aimed to explore the associations of histologic chorioamnionitis with qualitative/quantitative brain MRI measures at term-equivalent age in preterm infants. We analysed conventional MR images using a modified version of a published reporting system, and we measured brain tissue compartment volumes using an automated method [13, 14].

We hypothesised that preterm infants with histological evidence of chorioamnionitis would have more brain injury at term-equivalent age as evidenced by white matter signal abnormalities, and smaller, less mature brains at term equivalent age compared with those without histologic chorioamnionitis.

## Methods

### Participants

Data were derived from two prospective longitudinal cohort studies of growth and development in preterm infants. Both studies comprised preterm infants born at or transferred to the Royal Women’s Hospital neonatal unit, Melbourne, which is a tertiary neonatal unit located within a large perinatal centre. The first study comprised MLPT infants (born between 32^+ 0^ and 36 weeks’ gestation [13]), and the second comprised infants born at less than 30 weeks’ gestation [15]. These cohorts were selected to answer different research questions; there was no deliberate attempt to exclude infants born at 30 or 31 weeks’ gestation. Exclusion criteria did include genetic or congenital problems known to cause long-term developmental problems (Additional file 1).

Extensive perinatal data were collected by chart review. Birthweight was converted to Z-scores using LMS growth reference charts [14].

The Human Research Ethics Committees of the Royal Women’s Hospital and the Royal Children’s Hospital, Melbourne, approved both studies. Written informed consent was obtained from the parents of all participants. Details of the contact and tracing of the cohorts have previously been described [13, 15].

### Placental pathology

Where available, placentas were sent to the Pathology Department at the Royal Women’s Hospital for microscopic/macroscopic analysis. Placental histopathology is performed as part of clinical practice for preterm births. Samples for histopathological examination were fixed in formalin, processed with tissue sections cut to 3-5 mm thickness and stained with haematoxylin and eosin. The placentas were then examined microscopically.

A single investigator (CG) reviewed the histopathology reports and classified the placentas into those with evidence of inflammation (maternal, fetal or both) and those without. Where there was uncertainty in the reports, an anatomical pathologist (JP) reviewed the reports.

### Magnetic resonance imaging (MRI) scan

An MRI brain scan was undertaken at term equivalent age using previously published techniques [13, 15]. MRI scans of the brain were performed using a 3 T Magnetom Trio Tim system (Siemens, Erlangen, Germany) during natural sleep. Conventional T1-weighted and T2-weighted axial images were used for the analysis of this study.

A modified MRI scoring system based on a published system [13, 14] was used to assess MRIs. All images were reviewed by two experienced neuroradiologists (LC and CD), who were unaware of perinatal history. All MRI assessors had undergone similar training. Data were recorded on presence of brain injury (white matter signal abnormalities, periventricular cysts, intraventricular haemorrhage), brain maturation (degree of myelination of the posterior limb of the internal capsule, gyral maturation), brain size (brain biparietal diameter, corpus callosal measures of the genu, midbody and splenium, deep nuclear grey area and transcerebellar diameter) and cerebrospinal fluid measures (lateral ventricular size, interhemispheric distance). A global MRI score was also calculated based on an established scoring system using measures of brain injury, maturation and size as listed above [13]. Inter- and intra-rater reliability for items of this scoring system have been published previously [13]. Inter-rater reliability for MRI measures on 10 scans were generally high for all reported MRI metrics (0.82–0.98), with the exception of smaller measures like the genu of the corpus callosum (0.79) and the body of the corpus callosum (0.61). Kappa values of inter-rater reliability for categorical measures (degree of myelination of the posterior limb of the internal capsule and gyral maturation) were 1.0 [13].

### Infant brain segmentation

An automated Morphologically Adaptive Neonatal Tissue Segmentation (MANTiS) was used to classify the T2 structural MR image of the brain into the following structures: white matter, cortical grey matter, cerebrospinal fluid, subcortical gray matter (including deep nuclear gray matter, hippocampus and amygdala), brainstem, and cerebellum [16]. This technique extends the unified segmentation approach to tissue classification implemented in Statistical Parametric Mapping (SPM) software to infant brain scans. MANTiS combines unified segmentation, template adaptation via morphological segmentation tools and topological filtering. This technique reliably classifies the tissues even in the presence of brain abnormalities common in preterm infants, including enlarged ventricles and white matter signal intensity abnormalities. As the superior aspects of the extracerebral cerebrospinal fluid may not be included in all subjects, the volume calculations potentially underestimate the volumes of cerebrospinal fluid. Thus, we have not included cerebrospinal fluid volumes in the final analysis.

### Data analysis

Data were analysed using Stata version 14 (Stata Corporation, College Station, TX). Participant characteristics were compared between those included in this study (i.e with both placental histology data and MRI) with those not included using means and standard deviations (continuous variables) and proportions (categorical variables).

The relationships between histologic chorioamnionitis and MRI measures were explored using linear and logistic regression fitted to the MRI measures with histologic chorioamnionitis as a predictor. Models were fitted using generalised estimating equations with robust (sandwich) estimation of standard errors to allow for multiple births within a family. Results are presented firstly adjusted for gestation at MRI, and then adjusted for gestation at MRI as well as for gestation at birth, birthweight z-score, prolonged rupture of membranes, vaginal delivery, singleton birth, bronchopulmonary dysplasia (defined as oxygen requirement at 36 weeks’ corrected age), necrotizing enterocolitis and sepsis (culture positive or raised inflammatory markers and/or white cell count resulting in at least 5 days’ of antibiotics), all of which have been identified as potential confounders of this relationship in previous studies [2, 3, 5]. Results are presented as odds ratios (OR) and 95% confidence intervals (CI) for categorical variables, or mean differences and 95% CI for continuous brain measures.

## Results

Of the 349 preterm infants recruited into the 2 studies, 212 (61%) had both placental histology and MRI performed and are included in this study. Of these, 47 (22%) had histologic chorioamnionitis; 15 had maternal inflammatory response only, 31 had both maternal and fetal inflammatory response, and 1 had mild evidence of fetal inflammatory response only. All MRIs were suitable for qualitative assessment including brain metrics, but only 159 MRIs were suitable for volumetric analysis.

Compared with participants from the original birth cohorts who were not included in the current analysis, participants included in this study had lower mean birthweight z-scores (− 0.5 vs − 0.2) and were less likely to have received postnatal corticosteroids (3.2% vs 8.7%). The other characteristics were similar between those included in this study and those with incomplete data (data not shown). There were 159 participants with MRI suitable for segmentation. Participants with segmentable MRIs were similar in characteristics to those included in the study but who did not have MRI suitable for segmentation (data not shown).

Participant characteristics for the study sample (*n* = 212) are summarised in Table 1. Participants with histologic chorioamnionitis were born at earlier gestational ages and had a lower birthweight z-score than those without histologic chorioamnionitis. Participants with histologic chorioamnionitis were more likely to have had ruptured membranes for > 24 h, be singletons and be delivered by vaginal birth.

**Table 1:** Participant characteristics

	Histologic chorioamnionitis	
	Present (*n* = 47)	Absent (*n* = 165)
Gestation at birth in weeks, mean (SD)	29.8 (3.6)	32.2 (3.2)
Birthweight in grams, mean (SD)	1505 (728)	1686 (588)
Birthweight z-score, mean (SD)	−0.1 (0.9)	− 0.7 (1.1)
Gestation at MRI in weeks, mean (SD)	42.1 (1.5)	42.0 (1.4)
Antenatal corticosteroids	39 (83.0)	122 (73.9)
Ruptured membranes > 24 h	22 (46.8)	13 (8.2)
Vaginal delivery	29 (61.7)	36(21.8)
Male	24 (51.1)	85 (51.5)
Singleton	37(78.7)	85 (51.5)
Postnatal corticosteroids	2 (4.9)	4 (2.7)
Bronchopulmonary dysplasia^a^	9 (19.2)	15 (9.1)

Data are n (%) unless otherwise specified, *SD* Standard deviation, *PVL* Periventricular leukomalacia, *CI* Confidence interval. ^a^oxygen requirement at 36 weeks’ corrected age

Inter-rater reliability for MRI measures on 10 scans were generally high for all reported MRI metrics (0.82–0.98), with the exception of smaller measures like the genu of the corpus callosum (0.79) and the body of the corpus callosum (0.61). Kappa values of inter-rater reliability for categorical measures (degree of myelination of the posterior limb of the internal capsule and gyral maturation) were 1.0.

### Associations of histologic chorioamnionitis with brain MRI measures (Table 2)

Histologic chorioamnionitis was associated with increased odds of intraventricular haemorrhage and less mature gyral maturation. Both relationships disappeared following adjustment for confounders.

**Table 2:** Association between histologic chorioamnionitis and MRI measures

Histologic chorioamnionitis				
	Present (*n* = 47)	Absent (*n* = 165)	OR^a^ (95% CI) *p* value	Adjusted OR^b^ (95% CI) *p* value
(I) Brain injury				
Abnormal white matter signal, n (%)	13 (7.9)	4 (8.5)	1.2 (0.4, 4.0) *p* = 0.78	0.7 (0.2, 2.3) *p* = 0.59
Cystic periventricular leukomalacia, n (%)	1 (2.1)	0 (0)	–	–
Intraventricular haemorrhage, n (%)	9 (19.2)	5 (3.0)	7.4 (2.4, 23.1) *p* = 0.001	1.8 (0.5, 7.0) *p* = 0.41
(II) Brain maturation				
Complete myelination of the PLIC, n (%)	41 (87.2)	139 (84.2)	1.5 (0.3, 3.4) *p* = 0.58	0.9 (0.2, 4.4) *p* = 0.99
Gyral maturation< 38 weeks, n (%)	29 (61.7)	71 (43.0)	2.0 (1.0, 3.8) *p* = 0.05	1.6 (0.7, 3.6) *p* = 0.24
(III) Brain metrics (a) Brain measures			Coefficient^a^ (95% CI) *p* value	Adjusted coefficient ^b^ (95% CI) *p* value
Brain biparietal diameter (mm)	83.4 (4.1)	82.5 (4.5)	0.6 (−0.7, 1.9) *p* = 0.38	0.9 (− 0.6, 2.3) *p* = 0.23
Corpus callosum – genu (mm)	3.9 (1.5)	4.3 (1.4)	−0.3 (− 0.8, 0.1) *p* = 0.14	0.1 (− 0.4, 0.5) p = 0.82
Corpus callosum – body (mm)	2.0 (0.6)	2.1 (0.5)	−0.1 (− 0.3, 0.1) *p* = 0.45	0.1 (− 0.2, 0.2) *p* = 0.72
Corpus callosum – splenium (mm)	3.4 (1.2)	3.8 (1.0)	−0.3 (− 0.7, 0.1) *p* = 0.10	−0.1 (− 0.4, 0.3) *p* = 0.82
Deep nuclear gray matter (mm^2^)	1250.4 (104.1)	1235.6 (106.2)	8.9 (−25.0, 42.8) *p* = 0.61	12.7 (−22.7, 48.1) *p* = 0.48
Transcerebellar diameter (mm)	55.1 (2.9)	55.5 (3.1)	−0.6 (−1.5, 0.3) *p* = 0.20	− 0.6 (− 1.6, 0.3) *p* = 0.21
(b) Cerebrospinal fluid measures (mm)				
Right lateral ventricle	6.3 (2.5)	5.9 (1.8)	0.4 (−0.3, 1.1) *p* = 0.30	0.4 (−0.2, 1.1) *p* = 0.15
Left lateral ventricle	5.9 (2.4)	5.6 (1.8)	0.3 (−0.5, 1.0) *p* = 0.47	0.2 (−0.4, 0.9) *p* = 0.44
Interhemispheric distance	3.1 (1.8)	2.8 (1.3)	0.2 (−0.4, 0.8) *p* = 0.49	−0.4 (−1.0, 0.1) p = 0.14
(IV) Segmented brain volumes (cc)	n = 37	n = 122		
Total brain tissue	414.4 (37.9)	399.7 (43.9)	14.1 (1.9, 26.2) *p* = 0.02	6.4 (−10.1, 22.9) p = 0.45
Cortical grey matter	181.4 (17.4)	176.0 (20.8)	5.0 (−0.7, 10.8) *p* = 0.09	1.4 (−6.4, 9.2) *p* = 0.73
White matter	165.3 (15.7)	158.2 (17.4)	7.0 (1.8, 12.1) *p* = 0.008	4.4 (−2.4, 11.1) p = 0.20
Subcortical grey matter	31.9 (2.8)	31.1 (3.1)	0.7 (−0.3, 1.7) p = 0.15	0.1 (−1.0, 1.3) *p* = 0.83
Cerebellum	29.4 (3.2)	28.1 (3.6)	0.9 (0.1, 1.7) *p* = 0.04	0.1 (−1.2, 1.3) *p* = 0.93
Brainstem	6.5 (0.5)	6.3 (0.6)	0.2 (0.1, 0.4) *p* = 0.005	0.1 (−0.1, 0.3) *p* = 0.39
(V) Global MRI score	3.6 (2.8)	2.7 (2.3)	0.8 (−0.1, 1.7) *p* = 0.06	0.1 (−0.7, 0.7) *p* = 0.98

Results presented as mean (standard deviation) unless specified. *CI* Confidence interval, *OR* Odds ratio, *PLIC* Posterior limb of the internal capsule, *MRI* Magnetic resonance imaging. ^a^Adjusted for gestation at MRI; ^b^Adjusted for gestation at MRI, gestation at birth, birthweight z-score, ruptured membranes > 24 h, vaginal birth, singleton, bronchopulmonary dysplasia (oxygen requirement at 36 weeks’ corrected age), necrotizing enterocolitis and sepsis (culture positive or raised inflammatory markers and/or white cell count resulting in at least 5 days’ of antibiotics)

Histologic chorioamnionitis was also associated with larger total brain tissue volumes, white matter, cerebellar and brainstem volumes, although the mean differences in volumes were small (approximately 3–4%). Following adjustment for confounders, there was little evidence for relationships of histologic chorioamnionitis with brain segmented volumes.

There was little evidence for relationships of histologic chorioamnionitis with other parameters of brain injury, brain size or cerebrospinal fluid measures (using metric measurements).

## Discussion

This prospective cohort study of preterm infants identified associations between histologic chorioamnionitis and higher odds of intraventricular haemorrhage, less mature gyral maturation and slightly larger brain size. However, these relationships did not persist following adjustment for perinatal confounding variables suggesting the chorioamnionitis is not an independent predictor of brain injury or size at term equivalent age.

The association of chorioamnionitis with intraventricular haemorrhage has not been consistently demonstrated in previous studies [17]. The current study reported an OR of 1.7 for intraventricular haemorrhage in infants who had histologic chorioamnionitis, which disappeared after adjusting for confounders. This suggests that the association of chorioamnionitis with intraventricular haemorrhage was mediated by other perinatal confounders, such as gestational age at birth and birthweight z-score.

There is, however, more evidence in the literature supporting the associations of histologic chorioamnionitis with cystic periventricular leukomalacia and cerebral palsy, as reported in a recent meta-analysis [18]. A more recent study by Leviton et al [19] reported increased odds for ventriculomegaly and echolucent lesions on cranial ultrasound with histologic chorioamnionitis in infants with gestational ages < 28 weeks. In the current study we were unable to establish associations between histologic chorioamnionitis and cystic periventricular leukolamacia as our prevalence of cystic periventricular leukomalacia was low. Similarly, our ventricular measures were similar in infants with and without histologic chorioamnionitis. Of note, we measured ventricular size objectively, using MRI methods, rather than cranial ultrasound, as used in the study of Leviton et al. [19]

It is important to note that many of the early studies describing the association between chorioamnionitis (histologic or clinical) and brain injury used cranial ultrasound to report brain injury [20, 21]. Compared with MRI, cranial ultrasound is not sensitive at detecting non-cystic, multifocal white matter injury [1]. More recent studies using MRI to detect brain injury have found little evidence of associations for histologic chorioamnionitis with brain injury, development or growth at term equivalent age. Chau et al [22] studied a cohort of infants born between 24 and 32 weeks’ gestation using brain MRI. Infants with histologic chorioamnionitis were not at increased risk of white matter injury, abnormal brain metabolism as measured using magnetic resonance spectroscopy, or abnormal microstructural development. Reiman et al. reported on the relationship between histologic chorioamnionitis and regional brain volumes at term age in a cohort of very low birthweight (< 1500 g) or very preterm (< 32 weeks’ gestation) infants [23]. They were also unable to establish any relationships between histologic chorioamnionitis and brain volumes across several brain regions. A recently published study by Anblagan et al [24] looking at histological chorioamnionitis and altered brain microstructure as detected using diffusion MRI in infants < 32 weeks gestation found an association between the two. They concluded that their findings were suggestive of in utero brain inflammation in preterm infants exposed to histologic chorioamnionitis and it would be interesting to validate this finding in future analysis from our cohort. Although our study found a modest increase in brain volumes in those with histologic chorioamnionitis compared with those without, these differences were small and diminished after adjustment for confounding variables.

Data on the potential impact of chorioamnionitis on outcomes in MLPT infants is scarce. Lee et al [25] reported a higher rate of “adverse neonatal outcome” as defined by morbidities in the neonatal period in late preterm infants exposed to histologic chorioamnionitis compared with those who were not. Interestingly, there were no cases of brain injury on cranial ultrasound in either group. As such associations between histologic chorioamnionitis and brain injury, development and size in the MLPT population remain unknown.

Our study has several strengths. We used a prospective cohort of infants that included infants born < 30 weeks as well as those born MLPT among whom brain MRI data are scarce. In addition to advanced image analysis with brain segmentation, we used a simple, validated MRI assessment method [13, 15] suitable for clinicians caring for preterm babies. We used a definition of histologic chorioamnionitis rather than relying on reported clinical chorioamnionitis from obstetric histories.

We acknowledge the limitations as well. We used clinical reports for histology rather than a recognised classification system for case definition. However, these reports were done by a small number of experienced placental histologists at a single tertiary centre, and checked by a senior pathologist (JP) who is a co-author of the paper. In addition, not all participants in the original cohort studies had placental histology and MRI, and thus we may not have ascertained all cases of histologic chorioamnionitis within the cohort.

Further research directions include using advanced MRI techniques to probe white matter microstructure and brain metabolic profiles, which may be more sensitive than brain macrostructural characteristics in establishing associations between chorioamnionitis and brain injury in preterm infants. Correlation of neuroimaging findings with neurodevelopmental outcomes may provide further insight into the pathogenesis of neurodevelopmental deficits described in preterm children exposed to chorioamnionitis.

## Conclusions

In conclusion, this study found evidence that chorioamnionitis is associated with, but not an independent predictor of, IVH, less mature gyral maturation or brain volume. Other perinatal variables appear to contribute to the association between chorioamnionitis and preterm brain injury.

## Additional file

Additional file 1: Histologic chorioamnionitis in preterm infants: correlation with brain magnetic resonance imaging at term equivalent age. Supplemental files: Research questions. Copy of research questions used in study. (DOCX 53 kb)

## Abbreviations

CI: Confidence interval
IVH: Intraventricular haemorrhage
MANTiS: Morphologically Adaptive Neonatal Tissue Segmentation
MLPT: Moderate/late preterm
MRI: Magnetic resonance imaging
OR: Odds ratio
SPM: Statistical Parametric Mapping
