# Supplementary material for: Histologic chorioamnionitis in preterm infants: correlation with brain magnetic resonance imaging at term equivalent age
Source: BMC Pediatr. 2018 Feb 15;18:63. doi: 10.1186/s12887-018-1001-6 (PMC5815189; doi:10.1186/s12887-018-1001-6)
Supplement: Supplementary file 1 — Histologic chorioamnionitis in preterm infants: correlation with brain magnetic resonance imaging at term equivalent age. Supplemental files: Research questions. Copy of research questions used in study. (DOCX 53 kb) [file 12887_2018_1001_MOESM1_ESM.docx]

**Histologic chorioamnionitis in preterm infants: correlation with brain magnetic resonance imaging at term equivalent age**

**Supplemental files: Research questions**

What are the associations between histologic chorioamnionitis and brain injury, maturation and size as found on magnetic resonance imaging (MRI) of preterm infants at term equivalent age?

Clinical data

Presence or absence of chorioamnionitis on placental histology

MRI at term-equivalent age assessment

- intraventricular haemorrhage
- cysts, signal abnormalities)
- maturation (degree of myelination, gyral maturation)
- size of cerebral structures (metrics and brain segmentation)
